# Ubiquitylation-Mediated Fine-Tuning of DNA Double-Strand Break Repair

**DOI:** 10.3390/cancers12061617

**Published:** 2020-06-18

**Authors:** Barbara N. Borsos, Hajnalka Majoros, Tibor Pankotai

**Affiliations:** Department of Oral Biology and Experimental Dental Research, Faculty of Dentistry, University of Szeged, 83 Tisza L. Krt. H-6722 Szeged, Hungary; borsos.barbara.nikolett@gmail.com (B.N.B.); majoroshajnalka@gmail.com (H.M.)

**Keywords:** DNA repair, DSB, PTM, ubiquitylation, deubiquitylation

## Abstract

The proper function of DNA repair is indispensable for eukaryotic cells since accumulation of DNA damages leads to genome instability and is a major cause of oncogenesis. Ubiquitylation and deubiquitylation play a pivotal role in the precise regulation of DNA repair pathways by coordinating the recruitment and removal of repair proteins at the damaged site. Here, we summarize the most important post-translational modifications (PTMs) involved in DNA double-strand break repair. Although we highlight the most relevant PTMs, we focus principally on ubiquitylation-related processes since these are the most robust regulatory pathways among those of DNA repair.

## 1. Introduction

### Signaling through Ubiquitylation

Ubiquitylation is a reversible process, involving a cascade of E1 (ubiquitin-activating), E2 (ubiquitin-conjugating) and E3 (ubiquitin ligase) enzymes for ubiquitin addition and deubiquitylating enzymes (DUBs) for ubiquitin removal [1,2]. The enzyme cascade covalently links the small protein (76 amino acid residues) ubiquitin through its N-terminal glycine (G) residue to lysine (K) residues of the marked proteins.

Target proteins can be mono- or polyubiquitylated at different positions, leading to alternative responses that influence the stability of repair factors as well as their recruitment and localization. Although less information is known about monoubiquitylation, it has diverse effects on cellular processes. For instance, it plays a role in contributing to nuclear export-import, the chromatin recruitment or dissociation of certain proteins and also participates in protein degradation [3]. During polyubiquitylation, certain internal lysine residues (K6, K11, K27, K29, K33, K48 and K63) of ubiquitin serve as acceptor sites for the binding of additional ones [1]. According to these sites, proteins are marked for either participation in different cellular responses (e.g., K6, K27, K33 and K63) or for degradation by the 26S proteasome (e.g., K11 and K48).

Based on the type of DNA damage, different cellular processes and repair pathways can be activated, and these are tightly regulated by post-translational modifications (acetylation, methylation, phosphorylation, PARylation, SUMOylation, neddylation, ubiquitylation, etc.). Although, this is an extensively studied field, a comprehensive overview needs to be undertaken to enable a better understanding of the roles of these modifications in DNA double-strand break repair (DSBR).

In this review, we provide detailed information regarding the post-translational modification-mediated coordination of DNA repair processes. We present an overall summary of the ubiquitylation-related regulation of the most frequently induced DSBR pathways. Outlining the hitherto known tightly regulated steps will enable better understanding of the connection among them.

## 2. DNA Double-Strand Breaks

**Figure 1:**
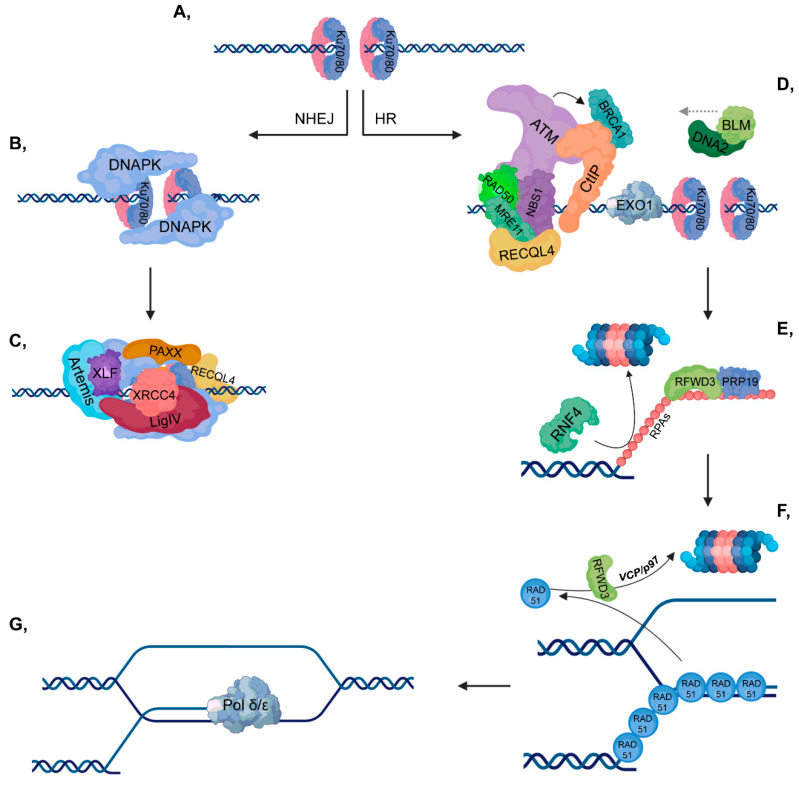
Double-strand break (DSB) repair pathways. (**A**) DNA double-strand break activates the binding of Ku70/80 heterodimers to the end of the broken DNA. (**B**–**C**) In non-homologous end joining (NHEJ), DNAPK is recruited to Ku70/80, then additional repair proteins and a ligase complex, which involves XLF-XRCC4-Ligase IV-PAXX also join. (**D**–**G**) In homologous recombination (HR), ataxia-telangiectasia mutated kinase (ATM) is recruited to the MRN complex (which consists of RAD50-MRE11-NBS1). CtIP and Exo1 are involved in short-range resection, while BLM-DNA2 participate in long-range resection. Replication protein A (RPAs) cover the ssDNA to protect it against nucleases and this step is stimulated by RFWD3 and PRP19, while RNF4 is involved in the ubiquitylation-mediated proteasomal degradation of RPAs. Then, the RPAs are replaced by RAD51 proteins to ensure the most suitable conformation for homology search. As a next step, RAD51 proteins are targeted for ubiquitin-mediated proteasomal degradation, in which RFWD3 and VCP/p97 are also involved. Pol δ/ε catalyzes the final extension step.

Upon serious DNA damage, double-strand break (DSB) formation activates two main repair pathways: non-homologous end-joining (NHEJ) and homologous recombination (HR). NHEJ, which is active throughout the cell cycle, is a faster but error-prone pathway, where the two DNA ends re-ligate with no or minimal homology sequence. The pathway consists of Ku70-80 heterodimer, DNAPK, XRCC4-XLF-PAXX and Ligase IV, as well as associated factors, and the DNA end processing is mediated by Artemis (Figure 1, left). On the other hand, HR is an error-free pathway, which requires longer resection and a RAD51-mediated sequence homology search between the broken DNA ends and the homologous sequence (Figure 1, right). Accordingly, HR occurs during the S and G2 phases when sister chromatids are available and serve as templates for repair. Additionally, activation of the DNA damage response (DDR) signal transduction pathway serves as a DNA damage sensor and also initiates an organized response to maintain cellular homeostasis.

### 2.1. DNA Damage Response

**Figure 2:**
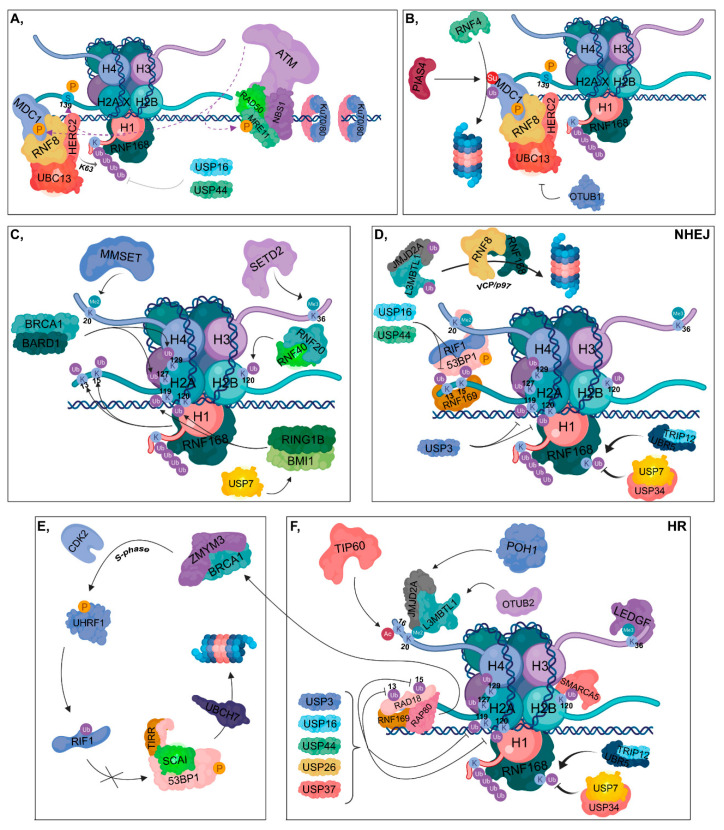
DNA damage response (DDR). (**A**) Ku70/80 heterodimers bind to broken DNA ends. The RAD50-NBS1-MRE11 complex is recruited resulting in the binding of ATM, which in turn phosphorylates MRE11, MDC1 and H2AX at Ser139. The RNF8-HERC2-UBC13 complex binds to phosphorylated MDC1. RNF8 is responsible for the K63-linked polyubiquitylation of H1, which can be reversed by the contribution of USP16 and USP44. RNF168 is recruited to polyubiquitylated H1. (**B**) PIAS4 SUMOylates MDC1, which is then ubiquitylated by RNF4, resulting in its proteasomal degradation. OTUB1 can inhibit UBC13, therefore hindering the DDR. (**C**) RNF168 catalyzes the ubiquitylation of H2A at its K13 and K15 residues, while BRCA1-BARD1 ubiquitylates H2A at K127 and K129. The RNF20-RNF40 is responsible for K120 ubiquitylation of H2B. RING1B-BMI1, stabilized by USP7, ubiquitylates H2A at K119 and K120. MMSET catalyzes H4K20 dimethylation and SETD2 trimethylates the H3K36 residue. (**D**) During NHEJ, RNF169 can bind to the ubiquitylated K13 and K15 lysine amino acids of H2A competing with the 53BP1-RIF1 complex to the same sites. However, these ubiquitins can be removed by USP16 and USP44. 53BP1-RIF1 can bind to H4K20me^2^ site as well. USP3 deubiquitylates H2A at K119 and K120. TRIP12 and UBR5 catalyze the ubiquitylation of RNF168, whose chain can be removed by USP7 and USP34. JMJD2A and L3MBTL1 are ubiquitylated by RNF8 and RNF168 and in turn degraded in the proteasome by a VCP/p97-dependent manner. (**E,F**) During HR, RNF169 can bind to K13 and K15 lysine amino acids of H2A competing with RAD18 and RAP80. Binding of RAP80 results in the recruitment of BRCA1 and ZMYM3. TIP60 acetylates H4 at K16, preventing the binding of 53BP1 to H4K20me^2^ site. JMJD2A and L3MBTL1 are deubiquitylated by POH1 and OTUB2, respectively. This step results in the recruitment of JMJD2A and L3MBTL1 to H4K20me^2^ site. Next, in the S phase, BRCA1 recruits CDK2, which then phosphorylates UHRF1 catalyzing the ubiquitylation of RIF1. TIRR is also involved in preventing 53BP1 binding to chromatin, and SCAI inhibits the binding of RIF1 to phosphorylated 53BP1, resulting in its UBCH7-mediated proteasomal degradation. USP3, USP16, USP44, USP26 and USP37 are involved in the deubiquitylation of H2A K13, K15, K119 and K120. LEDGF is recruited to the H3K36me^3^ site. Ubiquitylation of H2B at K120 allows the binding of SMARCA5 resulting in relaxed chromatin structure. TRIP12 and UBR5 catalyze the ubiquitylation of RNF168, whose ubiquitin molecules can be removed by USP7 and USP34.

**Figure 3:**
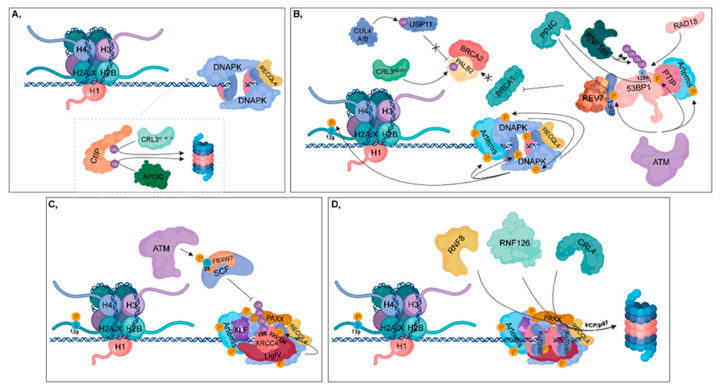
Non-homologous end joining (NHEJ). (**A**) During the G1 phase, CtIP is ubiquitylated by CRL3^KLHL15^ and APC/C, resulting in its proteasomal-mediated degradation. DNA-PK and unphosphorylated RECQL4 bind to Ku70/80 heterodimers, leading to the activation of NHEJ. (**B**) ATM phosphorylates 53BP1 at different sites, to which PTIP and RIF1 can bind (these sites can be dephosphorylated by PP4C in HR). PTIP binding is further enhanced by phosphorylated Artemis (catalyzed by ATM). RIF1 ensures the recruitment of REV7 to 53BP1, which inhibits the complex formation of BRCA1 with PALB2-BRCA2. In this process, CRL3^KEAP1^ ubiquitylates PALB2. This step is further supported by CUL4A/B, which attenuates the deubiquitylation of PALB2 through the ubiquitylation of USP11. Monoubiquitylation of 53BP1 at K1286 by RAD18 results in its retention, while polyubiquitylation at the same residue by RNF168 contributes to the recruitment of 53BP1 to the damaged site. At the break site, DNA-PK undergoes autophosphorylation and phosphorylates its target proteins, such as Artemis, Ku heterodimers and H2A.X at S139. (**C**) Following nuclease digestion and DNA synthesis, a ligase complex consisting of XLF-XRCC4-Ligase IV-PAXX can assemble. ATM phosphorylates the FBXW7 subunit of SCF at S26, which is responsible for polyubiquitylation of XRCC4 at K296. DNA-PK phosphorylates XRCC4 at S325/326. (**D**) Following ligation, Ku70/80 heterodimers are ubiquitylated by RNF8, RNF126 and CRL4, then degraded in the 26S proteasome by the contribution of VCP/p97.

**Figure 4:**
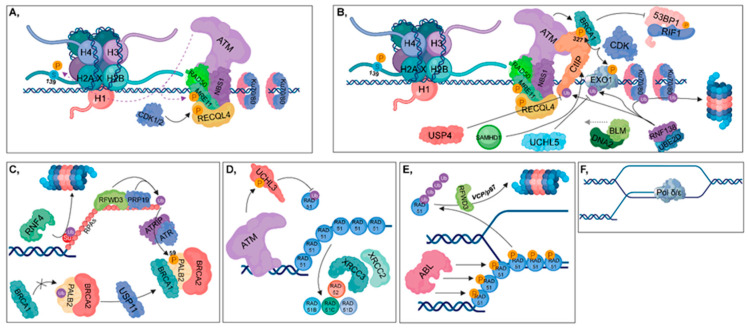
Homologous recombination (HR). (**A**) The RAD50-NBS1-MRE11 complex is recruited, resulting in the binding of ATM, which in turn phosphorylates MRE11, MDC1 and H2AX at Ser139. During the S and G2 phases, RECQL4 is phosphorylated by CDK1/2, contributing to its binding to MRE11, resulting in the activation of HR pathway. (**B**) CtIP (with the contribution of SAMHD1), BLM and DNA2 are recruited to the damaged site. MRE11 and CtIP take part in short-range end resection, while BLM and DNA2 are involved in long-range strand incision. CDKs phosphorylate CtIP and Exo1, while the RNF138-UBE2D complex is involved in their ubiquitylation. Deubiquitylation of CtIP and Exo1 is catalyzed by USP4 and UCHL5, respectively. The RNF138-UBE2D ubiquitylates Ku70/80 heterodimers, resulting in their proteasomal degradation. Phosphorylation of CtIP at S327 leads to the recruitment of BRCA1 (which is further stimulated by ATM) to that site, inhibiting 53BP1-RIF1 chromatin binding. (**C**) RFWD3 and PRP19 ubiquitylate RPAs, leading to the recruitment of ATRIP, which then induces the ATR-signaling pathway. Several factors are involved in facilitating the exchange of RPA for RAD51. First, PALB2 is deubiquitylated by USP11, allowing the formation of the BRCA1-PALB2-BRCA2 complex. ATR plays an important role during this process since it catalyzes the phosphorylation of PALB2 at S59. This step is crucial, since BRCA2 is required for RAD51 loading. RNF4 ubiquitin-ligase is involved in the proteasomal-dependent removal of the SUMOylated RPAs. (**D**) ATM phosphorylates and activates UCHL3, which in turn is capable of deubiquitylating RAD51. Furthermore, RAD51 loading is also enhanced by XRCC2, XRCC3, RAD52, RAD51B, RAD51C and RAD51D. (**E**) During postsynaptic filament formation, RAD51 should be removed from DNA. The DNA-binding affinity of RAD51 can be inhibited by its phosphorylation catalyzed by ABL. For complete removal, RFWD3-mediated polyubiquitylation of RAD51 is required, resulting in its VCP/p97-mediated proteasomal-dependent degradation. (**F**) Strand-extension is catalyzed by Pol δ/ε.

DDR initiates when Ku70/80 heterodimers bind to the ends of broken DNA to recruit repair proteins to the damaged site, depending on the repair pathway (Figure 2A). Based on its phosphorylation state, RECQL4 largely determines the type of repair pathway that should be activated. In the G1 phase, unphosphorylated RECQL4 binds to Ku70/80 heterodimers resulting in the activation of NHEJ (Figure 3A), while in the S and G2 phases, RECQL4 is phosphorylated by CDK1/2 promoting its binding to MRE11, therefore facilitating HR [4,5] (Figure 4A). CYREN (cell cycle regulator of NHEJ) plays a major role in the pathway that operates subsequent to the G1 phase. Binding of CYREN to Ku heterodimers at break sites with protruding ends inhibits the NHEJ pathway, ensuring the induction of error-free HR [6].

In the next step of DDR, the MRN (MRE11, RAD50 and NBS1) complex binds to the break site resulting in the recruitment of ataxia-telangiectasia mutated kinase (ATM), which phosphorylates MRN and H2A.X at S139 (referred to as γH2A.X), leading to mediator of DNA damage checkpoint protein 1 (MDC1) recruitment, which is then also phosphorylated by ATM [7]. The RNF8-HERC2 E3 ubiquitin ligase complex subsequently binds to phosphorylated MDC1. HERC2 promotes the complex formation of RNF8 with an E2 ubiquitin-conjugating enzyme, UBC13 [8,9,10]. RNF8 catalyzes K63-linked polyubiquitylation of H1 histones, facilitating the recruitment of an additional E3 ubiquitin ligase, RNF168 to ubiquitylated H1 histones [11] (Figure 2A). However, in U2OS cells it has been shown that at IR-induced foci, USP44 DUB can counteract the binding of RNF168 to the damaged site. Similar findings have been shown in the case of USP16, regardless of cell types, at IR-induced and FokI-induced DSB foci [12,13].

Among E3 ubiquitin ligases, SUMO-targeted ubiquitin ligases (STUbLs), such as RNF4 are involved in DDR, and through their SIM domain (SUMO-interacting motif) they can ubiquitylate SUMOylated proteins. RNF4 directs SUMOylated (catalyzed by PIAS4) MDC1 to undergo ubiquitin-mediated proteasomal degradation [14,15] (Figure 2B). In addition, RNF4 mediates UBC13-dependent K63-linked polyubiquitylation ensuring that both SUMOylated and ubiquitylated proteins can be recognized by RAP80 [16].

Various DUBs are also involved in DDR. OTU deubiquitinase, ubiquitin aldehyde binding 1 (OTUB1) deubiquitylates K48-linked chains and it contributes to the inhibition of UBC13 E2 ubiquitin-conjugating enzymes, thereby preventing DDR [17,18] (Figure 2B). Moreover, in U2OS cells, it has been demonstrated that OTUB1 can counteract with the RNF168-UBC13-mediated ubiquitylation at the damaged site. Consequently, OTUB1 presumably plays an important role in the coordination of RNF168 activity in the absence of DSBs [17]. Furthermore, OTUB1 interacts with and inhibits other E2 enzymes belonged to UBE2D and UBE2E families [17]. Through the inhibition of UBCH5 E2 enzyme, OTUB1 plays an important role in coordinating the stability of P53 [17,19]. Additional DUBs, such as OTU deubiquitinase, ubiquitin aldehyde binding 2 (OTUB2), the BRCA1/BRCA2-containing complex subunit 36 (BRCC36) and 26S proteasome-associated PAD1 homolog 1 (POH1) can hydrolyze K63-linked ubiquitin chains during DDR [20,21,22] (Figure 2F). Under physiological conditions, OTUB2 interacts with and removes the RNF8-generated ubiquitin chains from L3MBTL1. Additionally, as a response to DSB, in U2OS cells it has been shown that in the absence of OTUB2, the binding of 53BP1 and RAP80 to the repair foci is accelerated, which suggests a lower chance of HR pathway activation under these conditions [21].

RNF168 ubiquitylates H2A at K13 and K15, resulting in the recruitment of P53 binding protein 1 (53BP1), RNF169, RAP80 and RAD18 according to the appropriate pathway choice [23,24,25,26] (Figure 2C,D,F). 53BP1 contributes to NHEJ, RAP80 and RAD18 are involved in HR while RNF169 inhibits both repair pathways by competing with repair factors for K13 and K15 sites [27,28,29,30] (Figure 2D,F). Conversely, RNF169 is stabilized by one of its interaction partners, USP7, and that the RNF169-USP7 complex formation is required for facilitating HR [31]. Furthermore, RNF169 contributes to DNA end resection and in the absence of it, the malfunction of HR, single-strand annealing (SSA) and alternative non-homologous end-joining (aNHEJ) pathways can be observed [32]. RAP80 can localize to H2A K13 and K15 sites through its ubiquitin-interacting motifs (UIMs) leading to the recruitment of BRCA1 [33,34,35,36]. This step is also supported by the zinc finger protein zinc finger MYM-type containing 3 (ZMYM3), which interacts with BRCA1 and RAP80, thereby promoting HR [37] (Figure 2E,F). BRCA1-BARD1 ubiquitylates H2A at K127 and K129 contributing to the binding of SWI/SNF-related matrix-associated actin-dependent regulator of chromatin subfamily A containing DEAD/H box 1 (SMARCAD1) [38]. Additionally, H2A can be ubiquitylated at K119 and K120 by B-cell-specific Moloney murine leukemia virus integration site 1 (RING1B-BMI1) resulting in transcription silencing [39,40] (Figure 2C). However, ubiquitylation catalyzed by both BRCA1/BARD1 and RING1B/BMI results in gene silencing, each ligase complex is responsible for ubiquitylation of H2A at different residues, which lead to different molecular events. Although mutation of BARD1 results in malfunction of nucleosome binding, BMI mutants do not show the same phenomenon [41].

RNF168-mediated ubiquitylation of H2A can be regulated by the well-coordinated ubiquitylation- and deubiquitylation-modulated balance of RNF168 (Figure 2C). In this process, thyroid hormone receptor interactor 12 (TRIP12) and UBR5 E3 ubiquitin ligases as well as USP7 and USP34 DUBs are involved [42] (Figure 2D,F). USP7 stabilizes the RING1B-BMI1 complex, and USP3, USP16, USP26, USP37 as well as USP44 remove the ubiquitin chains from K13, K15, K119 and K120 residues of H2A [12,13,23,43,44] (Figure 2C,D,F). Nevertheless, it is important to note that USP26 and USP37 participate in the BRCA1-mediated HR pathway [43] (Figure 2E,F).

In addition to K13 and K15 sites of H2A, 53BP1 binds to H4K20me^2^ (catalyzed by MMSET methyltransferase) residues by the contribution of RNF8 and RNF168 [45] (Figure 2C,D). During NHEJ, these E3 ligases ubiquitylate 53BP1 inhibitory factors, such as Jumonji domain-containing protein A (JMJD2A also known as KDM4A) and lethal (3) malignant brain tumor-like protein 1 (L3MBTL1) facilitating the VCP/p97-linked proteasomal degradation of them. Thus, RNF8 and RNF168 directly promote the binding of 53BP1 to this modified chromatin site [46,47] (Figure 2D). Lethal (3) malignant brain tumor-like protein 2 (L3MBTL2) is responsible for the connection between RNF8 and RNF168. MDC1 has a potential role in the recruitment of L3MBTL2, which is subsequently ubiquitylated by RNF8, resulting in the binding of RNF168 to the DNA lesion [48].

In contrast, when HR is activated, JMJD2A and L3MBTL1 are deubiquitylated by POH1 and OTUB2 DUBs, respectively [21,22]. Additionally, TIP60 acetyltransferase, which catalyzes the transfer of acetyl-groups to K16 residue of H4, prevents the binding of 53BP1 to H4K20me^2^ sites by ensuring a more favorable environment for BRCA1 rather than for 53BP1 [49,50] (Figure 2F). Binding is further inhibited by Tudor-interacting repair regulator (TIRR), which interacts with the Tudor domain of 53BP1, through which it can bind to chromatin [51].

Suppressor of cancer cell invasion (SCAI) competes with replication timing regulatory factor 1 (RIF1) for the phosphorylated 53BP1 [52]. RIF1 is ubiquitylated by ubiquitin-like containing PHD and RING finger domains 1 (UHRF1) E3 ubiquitin ligase, which was previously phosphorylated by CDK2 in the S phase and recruited to DSB sites by BRCA1 [53,54]. 53BP1 is degraded by UBCH7 (also known as UBE2L3) through an E2-mediated proteasomal manner [55]. These steps contribute to the inhibition of the NHEJ pathway and activation of HR, thereby authorizing an error-free repair pathway choice (Figure 2E).

The heterodimeric ring finger protein 20-ring finger protein 40 (RNF20-RNF40) E3 ubiquitin ligase complex ubiquitylates H2B at K120 to promote chromatin relaxation through the recruitment of SWI/SNF-related matrix-associated actin-dependent regulator of chromatin A5 (SMARCA5), allowing the binding of repair factors involved in HR to the break site [56,57]. In addition to ubiquitylation, SET domain containing 2 (SETD2)-mediated trimethylation of H3K36 promotes the binding of lens epithelium-derived growth factor (LEDGF), which then facilitates recruitment of CtBP-interacting protein (CtIP) to the break site resulting in the activation of HR pathway [58] (Figure 2C,F).

### 2.2. Non-Homologous End-Joining

During the G1 phase, CtIP is degraded by the ubiquitin-proteasome system, in which anaphase promoting complex/cyclosome (APC/C) and Cullin-RING E3 ubiquitin ligase 3 Kelch-like family member 15 (CRL3^KLHL15^) E3 ligases are involved [59,60] (Figure 3A). DNA-dependent protein kinase (DNA-PK) and Artemis endonuclease are recruited to the break sites, where Ku70/80 heterodimers have already bound [61,62] (Figure 3A,B). ATM phosphorylates 53BP1 at different sites, to which its downstream mediators, PAX transactivation domain-interacting protein (PTIP) and RIF1 can bind [63,64].

Artemis is also phosphorylated by ATM, contributing to its binding to PTIP. RIF1 ensures the binding of REV7, which interferes with HR by inhibiting the binding of BRCA1 to PALB2 [65,66]. Moreover, CRL3^KEAP1^ E3 ligase is involved since it ubiquitylates PALB2. This step is further supported by CUL4A/B E3 ligase, which in G1 phase ubiquitylates USP11 DUB responsible for removing ubiquitin groups from PALB2 [67]. According to the type of 53BP1 ubiquitylation, opposite pathways can be activated: (i) monoubiquitylation at K1286 by RAD18 might play a role in its retention, thereby inducing HR, and (ii) polyubiquitylation by RNF168 at the same residue contributes to the recruitment of 53BP1 to the break sites, promoting NHEJ [68,69] (Figure 3B).

At DSBs, DNA-PK undergoes autophosphorylation, which activates its kinase activity specifically for substrates, such as Ku proteins, Artemis and H2A.X, in close proximity to the break [70] (Figure 3B). Phosphorylation of Ku proteins leads to their reduced DNA-binding affinity by promoting their inward translocation from DNA ends to allow other NHEJ factors to access the break [71,72]. DNA-PK phosphorylates ATM at inhibitory sites, thereby preventing the activation of HR [73]. Artemis phosphorylation catalyzed by DNA-PK leads to the dissociation of its C-terminal inhibitory region resulting in its activation [61,74]. However, Artemis has DNA-PK-dependent 5′-3’ and 3′-5′ endonuclease activities, it preferentially generates 4-nt 3′ overhangs, thereby reducing the probability that RPA will bind to RAD51 [75]. Additionally, Artemis also possesses a DNA-PK-independent 5′-3′ exonuclease activity and a recently described X-ray repair cross complementing 4 (XRCC4)-Ligase IV-dependent 3′-5′ endonuclease activity [76].

During NHEJ, DNA Pol µ and Pol λ catalyze accurate DNA synthesis [77,78]. These polymerases possess a BRCA1 C-terminus (BRCT) domain through which they can interact with Ku proteins [79]. Pol µ and Pol λ can incorporate nucleotides template-independently and template-dependently, respectively [80]. After nuclease digestion and DNA synthesis, a ligase complex (XLF-XRCC4 Ligase IV and PAXX) can assemble. Ligase IV interacts with Artemis and Ku proteins, while XRCC4 binds to XRCC4-like factor (XLF) and Ligase IV [81]. Ligase IV harbors two BRCT domains through which it can bind to two Ku proteins at each end of DNA [82]. Polyubiquitylation of XRCC4 at K296 mediated by SCF^FBXW7^ promotes its connection with Ku proteins. This process is regulated by two kinases, since ATM phosphorylates FBXW7 at S26 and DNA-PK catalyzes the phosphorylation of XRCC4 at S325/S326 [83]. In addition, XLF and paralog of XRCC4 and XLF (PAXX) also bind to Ku heterodimers [84] (Figure 3C). When ligation has been completed, Ku heterodimers are ubiquitylated by RNF8, CRL4 or RNF126, then degraded by the 26S proteasome with the contribution of VCP/p97 segregase [85] (Figure 3D).

Several accessory proteins are involved in NHEJ, such as polynucleotide kinase (PNK) and Aprataxin. Unlike most kinases, PNK has both kinase and phosphatase activities. Its kinase activity is needed for the 5′ phosphate group addition, while undesired phosphate groups from the 3′ end can be removed by its phosphatase activity [86]. Aprataxin is a deadenylase, which is capable of resolving a Ligase IV-mediated incomplete ligation, when an AMP group still remains at the 5′ end of DNA [87]. These two accessory proteins are not required for NHEJ, except when repair cannot be properly completed [88].

### 2.3. Homologous Recombination

HR can be divided into presynaptic, synaptic and postsynaptic phases. During the presynaptic phase, RAD51 molecules are loaded onto the ssDNA leading to presynaptic filament formation. ssDNA is stretched, which ensures a rapid homology search. In the synaptic phase, RAD51 contributes to the heteroduplex (D-loop or Holliday junction) formation by facilitating the pairing between the invading DNA strand and the homologous sequence. In the postsynaptic phase, RAD51 proteins dissociate from DNA, allowing the subsequent DNA synthesis and ligation.

#### 2.3.1. Presynaptic Phase/Filament Formation

As described in the DDR section, the MRN complex is the first sensor in double-strand break recognition [89]. Thus, ATM is recruited to the break site and phosphorylates MRE11, H2A.X and MDC1 [10]. Phosphorylation of H2A.X at S139 (γH2A.X) is one of the main signatures of DNA double-strand breaks [7] (Figure 2A and Figure 4A).

Subsequently, phosphorylated MDC1 binds to γH2A.X recruiting nucleases, such as CtIP, Exonuclease 1 (Exo1) and DNA2 in complex with Bloom syndrome protein (BLM) helicase (BLM-DNA2) to initiate DNA end resection [7,90,91]. CtIP can also be recruited to the break site by SAM and HD domain containing deoxynucleoside triphosphate (SAMHD1) dNTP tri-phosphohydrolase (Figure 4B) [92]. MRN (via the 3′-5′ endonuclease activity of MRE11) and CtIP (via its 5′-3′ endonuclease activity) are involved in short-range end resection, while Exo1 (via its 5′-3′ exonuclease activity) and BLM-DNA2 (catalyzes only 5′ strand incision) mediate long-range strand incision [90,91].

In the S and G2 phases, CDKs phosphorylate CtIP and Exo1, thereby strengthening the HR pathway choice [93,94]. In contrast, the RNF138 (E3) in complex with UBE2D (E2) catalyzes the ubiquitylation of CtIP and Exo1 retaining CtIP at the break sites, while it initiates the ubiquitin-mediated proteasomal degradation of Ku heterodimers [95,96] (Figure 4B). Furthermore, MRE11 (via its 3′-5′ exonuclease activity) contributes to the removal of Ku heterodimers from the damaged site [97]. USP4 and UCHL5 DUBs hydrolyze inhibitory ubiquitin chains from CtIP and Exo1, respectively [98,99] (Figure 4B). In addition, USP4 interacts with MRN and CtIP promotes the interaction between these proteins [98,100]. Nevertheless, phosphorylation of CtIP at S327 ensures a proper binding surface for BRCA1 recruited by ATM [101,102] (Figure 4B). BRCA1 is capable of restraining 53BP1 binding to DNA break sites through the recruitment of UHRF1, which ubiquitylates RIF1, one of the binding partners of 53BP1 [53] (Figure 2E and Figure 4B). This inhibition is enforced by PP4C phosphatase that dephosphorylates 53BP1 (Figure 3B) [54].

Protruding ssDNA ends are protected from nucleases by heterotrimeric Replication protein A (RPAs consisting of RPA70, RPA32 and RPA14). In this process, RING finger and WD repeat domain 3 (RFWD3) and PRP19 play a major role, since they interact with and monoubiquitylate RPAs [103], leading to the recruitment of ATRIP, which then induces the ATR-signaling pathway (Figure 4C).

In the next step, RPAs should be exchanged with RAD51 nucleo-protein filaments, which is a crucial step in HR, since RAD51 is indispensable for homology searching and strand invasion. First, RNF4 STUbL ubiquitylates the SUMOylated RPAs targeting them for proteasomal degradation [14]. Additionally, BRCA2 also promotes RAD51 loading through a well-regulated process. BRCA1 recruits BRCA2 to the break site through its interaction with the BRCA2 binding partner, partner and localizer of BRCA2 (PALB2) [104]. To promote their interaction, PALB2 should first be deubiquitylated by USP11 resulting in the formation of a complex composed of BRCA2-PALB2-BRCA1. Furthermore, ATR-mediated phosphorylation of PALB2 at S59 also enhances the binding of BRCA1 to the PALB2-BRCA2 complex [105] (Figure 4C). 

Loading of RAD51 is also regulated by UCHL3 phosphorylated and activated in an ATM-dependent manner, which enables UCHL3 to deubiquitylate RAD51 [106] (Figure 4D). RAD54 plays a dual role in the regulation of nucleo-protein filament formation since it is involved in either the loading or displacement of RAD51. RAD54 interacts with RAD51 through its N-terminal region and binds to dsDNA through its RecA domain. Thus, RAD51 monomers can be either loaded onto DNA by the contribution of RAD54 or displaced from dsDNAs by the translocation of RAD54 [107]. Following RAD51 loading, the presynaptic filament is formed, comprising of six RAD51 monomers and 18 nucleotides per helical turn [108].

#### 2.3.2. Synaptic Phase/Homology Search and Postsynaptic Phase/Strand Invasion

During the synaptic phase, RAD51 is the key factor that is responsible for D-loop formation between the invading DNA strand and its homologous pair. RAD51 loading is promoted by RAD52 and RAD51 paralogues, such as RAD51B/C/D (RAD51 homolog 2, 3, 4), X-ray repair cross complementing 2 (XRCC2) and X-ray repair cross complementing 3 (XRCC3) (Figure 4D). These factors ensure the stability of presynaptic filament and protect RAD51 from negative regulators, such as Fanconi anemia group M protein (FANCM), BLM, regulator of telomere helicase 1 (RTEL1) or PCNA-interacting partner (PARI) helicases. RAD52 interacts with RAD51, thereby promoting its loading onto ssDNA although it is not directly involved in the removal of RPA from DNA [109,110,111,112]. Negative regulators also play an important role in HR since they contribute to the maintenance of genome instability. For instance, D-loop formation can be deleterious at stalled replication forks, and nucleo-protein intermediates may induce cell cycle arrest or apoptosis [113].

RAD51 may inhibit postsynaptic filament formation since it could impede DNA polymerases (Pol δ/ε). To reconcile recombination intermediates and chromatin assembly, RAD51 should be entirely removed from the 3′ end of the invading DNA strand. Phosphorylation of RAD51 by c-ABL prevents its DNA-binding [114]. To completely remove RAD51, it is polyubiquitylated by RFWD3, resulting in its VCP/p97-mediated proteasomal degradation [115] (Figure 4E,F). Finally, resolvase and ligase are involved in the removal of branched intermediates and gap closing in DNA strands, respectively.

## 3. Conclusions

To preserve genome integrity, cells have developed cellular responses and repair processes to eliminate DNA lesions. For efficient and proper repair, each protein involved in the elimination of DNA damage must be tightly regulated in a precise spatiotemporal manner. Uncontrolled stability, failure in the regulation of subcellular localization and the catalytic activity of repair proteins can lead to an inaccurate repair pathway, eventually resulting in cancerous malformations. In recent years, several research groups have investigated the precise coordination of repair factors activated by different kinds of DNA damage. If we understand the fine-tuning of DNA repair, this could contribute to the invention of more efficient therapeutic compounds. According to the current repair pathway, different E3 ligases are involved in the activation of downstream repair factors. Among these E3 ligases, the main ubiquitin modifiers during DNA damage-induced ubiquitin signalization may serve as potential therapeutic targets: RNF8 and RNF168 are implicated as crucial components of DDR resulting in the activation of either HR or NHEJ pathway. These E3 ligases play a pivotal role in the precise regulation of tumor suppressor genes and proto-oncogenes. Moreover, inappropriate coordination of the level of tumor suppressors and proto-oncogenes can lead to cancerous malformations. Therefore, the physiological level of regulatory E3 ligases needs to be tightly coordinated. According to the tumor type, personalized tumor therapy can be achieved through the invention of either agonists or antagonists of the targeted E3 ligase; thus, novel anti-tumor agents can be developed, which might relieve the side-effects during therapy.

Since ubiquitylation is a reversible process, in addition to E3 ligases, DUB enzymes also have specific regulatory functions in the repair processes. It has been comprehensively established that ubiquitin signaling is crucial for the recruitment of downstream repair proteins to the damaged site, which indicates that they should be spatiotemporally modulated by ubiquitin-dependent proteasomal degradation.

Our knowledge regarding damage-induced ubiquitin signaling has been rapidly increasing, and recognition of the more precise role of DNA repair proteins in tumorigenesis, neurodegeneration and other serious diseases should be more accurately clarified. Moreover, the discovery of novel factors that influence DNA repair will enhance our understanding of the tightly orchestrated regulation of DNA repair proteins.
